# Implications of Altered Glutathione Metabolism in Aspirin-Induced Oxidative Stress and Mitochondrial Dysfunction in HepG2 Cells

**DOI:** 10.1371/journal.pone.0036325

**Published:** 2012-04-30

**Authors:** Haider Raza, Annie John

**Affiliations:** Department of Biochemistry, Faculty of Medicine and Health Sciences, United Arab Emirates (UAE) University, Al Ain, United Arab Emirates; Faculty of Pharmacy, Ain Shams University, Egypt

## Abstract

We have previously reported that acetylsalicylic acid (aspirin, ASA) induces cell cycle arrest, oxidative stress and mitochondrial dysfunction in HepG2 cells. In the present study, we have further elucidated that altered glutathione (GSH)-redox metabolism in HepG2 cells play a critical role in ASA-induced cytotoxicity. Using selected doses and time point for ASA toxicity, we have demonstrated that when GSH synthesis is inhibited in HepG2 cells by buthionine sulfoximine (BSO), prior to ASA treatment, cytotoxicity of the drug is augmented. On the other hand, when GSH-depleted cells were treated with N-acetyl cysteine (NAC), cytotoxicity/apoptosis caused by ASA was attenuated with a significant recovery in oxidative stress, GSH homeostasis, DNA fragmentation and some of the mitochondrial functions. NAC treatment, however, had no significant effects on the drug-induced inhibition of mitochondrial aconitase activity and ATP synthesis in GSH-depleted cells. Our results have confirmed that aspirin increases apoptosis by increased reactive oxygen species production, loss of mitochondrial membrane potential and inhibition of mitochondrial respiratory functions. These effects were further amplified when GSH-depleted cells were treated with ASA. We have also shown that some of the effects of aspirin might be associated with reduced GSH homeostasis, as treatment of cells with NAC attenuated the effects of BSO and aspirin. Our results strongly suggest that GSH dependent redox homeostasis in HepG2 cells is critical in preserving mitochondrial functions and preventing oxidative stress associated complications caused by aspirin treatment.

## Introduction

Inflammation induced response have been implicated in the pathogenesis of numerous diseases including cancer. Increased formation of inflammatory cytokines such as TNF-α, IL-1β and others has been described in the pathophysiology of degenerative diseases, infections and drug-induced toxicities [1], [2]. Arachidonic acid metabolism to prostaglandins by cyclooxygenase (COX) is a key for initiation of many inflammatory responses [3], [4]. Non-steroidal anti-inflammatory drugs (NSAIDs), including aspirin (ASA) reduce inflammation by inhibiting the synthesis of prostaglandins (PGs) and induce apoptosis in a variety of cancer cells [5], [6], [7].Tumor cells are known to develop resistance towards therapeutic drugs and irradiation due to inhibition of apoptotic stimuli in these cells. NSAIDs have been suggested to induce apoptosis in resistant tumor cells [8]. However, the precise molecular mechanisms by which these compounds induce apoptosis and promote antitumor action are not clearly understood. The most important and best defined molecular target for ASA is COX. However, there are multiple reports suggesting several additional mechanisms of action, independent of their ability to inhibit COX activity, that may contribute to its anti-cancer and anti-inflammatory effects [9],[10],[11].There is little information on the selectivity and specificity of NSAID-mediated effects and therefore a better understanding of the molecular and biochemical mechanisms for aspirin and other NSAIDs is essential for therapeutic use of drugs in multiple disorders associated with inflammation. Concerns about the selectivity of NSAIDs and associated toxicity have limited the widespread use of this drug. Recent epidemiological studies on humans and experimental models in diabetes, cancer and cardiovascular diseases have demonstrated that regular use of ASA alone or as an adjuvant may improve the outcome of disease prevention/protection in favor of benefit: risk ratio [11].

Aspirin has been shown to exert its cytotoxicity and anti-inflammatory effects through multiple mechanisms of action that may include generation of reactive oxygen species, increased oxidative stress, mitochondrial dysfunction and induction of apoptosis [6], [12], [13]. However, aspirin has also been shown to protect endothelial cells from oxidative damage via nitric oxide/cGMP pathway [14]. Aspirin has also been shown to protect against acetaminophen-induced liver toxicity due to down regulation of proinflammatory cytokines rather than COX-1 inhibition [15]. Alteration in innate immune response by Tlr9 and the Nalp3 inflammasome in acetaminophen-induced hepatotoxicity and induction of autophagy through the removal of damaged mitochondria and oxidative stress may be the potential mechanisms for aspirin-induced cytoprotection [15], [16]. Mitochondrial oxidative stress and respiratory dysfunctions in cancer cells may, therefore, lead to the activation of apoptotic signals by the release of apoptosis-inducing factors and proteins and subsequent activation of the caspases [17], [18], [19]. A group of compounds, termed ‘mitocans’, which target the mitochondrial structural integrity and respiratory and thiol redox functions, are being studied extensively, as they have a potential to be effective therapeutic drugs against cancer [20], [21], [22]. Recently, we have demonstrated that dose- and time-dependent ASA treatment of HepG2 cells caused cell cycle arrest, increase in ROS production, reduction in mitochondrial potential, and alterations in mitochondrial redox and respiratory functions [23]. Based on the mitochondrial toxicity in cancer cells, ASA may also be classified as a potential ‘mitocan’. Our present hypothesis is that compounds which can directly target mitochondrial function offer the advantage of mitochondrial-mediated cell death independent of upstream signaling molecules that are impaired in cancer development, treatment and/or drug resistance. Therefore, in this study, we have used human hepatoma HepG2 cancer cells, a commonly used cell culture model, which has been treated with buthionine sulfoximine (BSO), a GSH depleting agent and N-acetylcysteine (NAC), a precursor of GSH synthesis, to study the implications of altered glutathione metabolism in ASA-induced oxidative stress, redox homeostasis and mitochondrial dysfunctions.

## Materials and Methods

### Chemicals

Aspirin (acetylsalicylic acid, ASA), cytochrome c, reduced and oxidized glutathione, (GSH and GSSG), 1-chloro 2,4-dinitrobenzene (CDNB), cumene hydroperoxide, glutathione reductase, 5,5′-dithio bis-2-nitrobenzoic acid (DTNB), 2-thiobarbituric acid, buthionine sulfoximine (BSO), N-acetylcysteine (NAC), NADH, NADPH, coenzyme Q2, antimycin A, dodecyl maltoside and ATP bioluminescent somatic cell assay kit were purchased from Sigma-Aldrich Fine Chemicals (St Louis, MO, USA). 2′, 7′- Dichlorofluorescein diacetate (DCFDA) was from Molecular Probes (Eugene, OR, USA). Aconitase assay kit was procured from Oxis International Inc. (Portland, OR, USA). Apoptosis detection kit for flow cytometry was from BD Pharmingen (BD Biosciences, San Jose, USA). Kits for caspase-3 and mitochondrial membrane potential assays were purchased from R&D System, MN, USA. HepG2 cells were purchased from American Type Culture Collection (Manassas, VA, USA) and have been used for studies in our recent publication [23]. Polyclonal antibodies against beta-actin, caspase-3, PARP, cytochrome c, Bcl-2, Tom-40 and FITC-conjugated secondary antibodies were purchased from Santa Cruz Biotechnology Inc. (Santa Cruz, CA, USA). Glutathione S-transferase A4-4 (GSTA4-4) antibody was a generous gift from Prof. Bengt Mannervik, Uppsala University, Stockholm, Sweden. Reagents for cell culture and for SDS-PAGE and Western blot analyses were purchased from Gibco BRL (Grand Island, NY, USA) and from Bio Rad Laboratories (Richmond, CA, USA) respectively.

### HepG2 cell culture, treatment and cellular fractionation

HepG2 cells were grown in poly-L-lysine coated 75 cm^2^ flasks (∼2.0–2.5×10^6^cells/ml) in DMEM medium supplemented with 1% nonessential amino acids, 2 mM glutamine, 10% heat inactivated fetal bovine serum in the presence of 5% CO2–95% air at 37°C as described before [23]. Cells were treated with 5 or 10 µmol/ml ASA (dissolved in 1 M Tris-HCl (pH 7.6) to a stock solution of 1 M and adjusted to pH 7.45 with 4 N HCl) for 48 hours. Selection of dose and time points for the test compounds were based on our previous publication [23] and literature search. The GSH level was depleted in HepG2 cells by the treatment of BSO, a known inhibitor of GSH synthesis, as described before [24]. Briefly, cells were treated with 100 µM BSO (dissolved in culture medium) for 18 h and washed with fresh serum free DMEM followed by aspirin treatment as above. At this concentration and time point, BSO alone has no significant effect on HepG2 cell viability (data not shown). In another set of experiments, ASA treatments (5 or 10 µmol/ml ASA 48 h) were performed in GSH-depleted cells (BSO treatment for 18 h) which were then treated with 10 mM NAC (dissolved in DMSO, then diluted with cell culture medium to the required concentration keeping the DMSO concentration not more than 0.1%) for 2 hr before the addition of ASA for 48 h to manipulate cellular GSH pools. Control cells were treated with vehicle alone (final DMSO concentration not more than 0.1% which has no significant effect on cellular functions and viability). After the desired time of treatment, cells were harvested, washed with PBS (pH 7.4) and homogenized in H-medium buffer (70 mM sucrose, 220 mM mannitol, 2.5 mM HEPES, 2 mM EDTA and 0.1 mM phenylmethylsulfonylfluoride, pH 7.4) at 4°C. Mitochondria and post mitochondrial (PMS) fractions were prepared by centrifugation and the purity of the isolated fractions was checked for cross contamination as described before [23].

### Measurement of GSH concentration

HepG2 cells were treated with BSO and/or NAC as described above and then exposed to different concentrations of ASA for 48 h. Sub cellular GSH levels in the mitochondria and PMS were measured by the NADPH-dependent GSSG-reductase catalyzed conversion of GSSG to GSH as described before [23], [25].

### ROS measurement and apoptosis by flow cytometry

The intracellular production of ROS was measured using the cell permeable probe DCFDA, which preferentially measures peroxides. Briefly, GSH-depleted cells (∼2×10^6^ cells/ml) treated with ASA with or without NAC were incubated with 5 µM DCFDA for 30 minutes at 37°C. Cells were washed twice with 1× PBS, trypsinized and resuspended in 3 ml of PBS and the fluorescence was immediately read on a Becton Dickinson FACScan with Cell Quest software (BD Biosciences, San Jose, USA).

The apoptosis assay using flow cytometry was performed according to the vendor's protocol (BD Pharmingen, BD Biosciences, San Jose, USA). Briefly, GSH-depleted cells treated with ASA with or without NAC were trypsinized, washed in PBS and resuspended (1×10^6^ cells/ml) in binding buffer (10 mM HEPES, pH 7.4, 140 mM NaCl, 2.5 mM CaCl_2_). A fraction (100 µl/1×10^5^ cells) of the cell suspension was incubated with 5 µl Annexin V conjugated to FITC and 5 µl propidium iodide (PI) for 15 mins at 25°C in the dark. 400 µl of binding buffer was added to the suspension and apoptosis was measured immediately using a Becton Dickinson FACScan analyzer. The apoptotic cells were estimated by the percentage of cells that stained positive for Annexin V-FITC while remaining impermeable to PI (AV+/PI−). This method was also able to distinguish viable cells (AV−/PI−) and cells undergoing necrosis (AV+/PI+).

### Measurement of DNA laddering

HepG2 cells were seeded on to 35 mm diameter culture dishes. After allowing a 24 h period for attachment, GSH-depleted cells were treated with 5 or 10 µmol/ml ASA for 48 h and NAC in some cases as described above. Total DNA was isolated and separated on 1.5% agarose gel. DNA fragmentation was visualized by UV transillumination after staining the electrophoretically separated fragments with 0.5 µg/ml ethidium bromide as described before [23].

### Measurement of GSH metabolism

HepG2 cells were treated with BSO with or without NAC as described above and then exposed to different concentrations of ASA for 48 h. Glutathione S-transferase (GST) activity using CDNB and glutathione peroxidase (GSH-Px) activity using cumene hydroperoxide as respective substrates were measured by standard protocols as described before [25].

### Measurement of lipid peroxidation

NADPH-dependent-membrane lipid peroxidation in ASA treated cells after BSO/NAC treatment was measured as thiobarbituric acid reactive substances (TBARS) using malonedialdehyde as standard as described previously [23].

### Assay of caspase-3 activity

GSH depleted HepG2 cells with or without NAC (2×10^6^ cells/well) were treated with different concentrations of ASA as described above and the caspase-3 substrate, DEVD-pNA was used to assay the protease activity colorimetrically as described in the vendor's protocol (R & D Systems).

### Measurement of mitochondrial membrane potential (MMP)

GSH depleted HepG2 cells with or without NAC (2×10^6^ cells/well) were treated with different concentrations of ASA as described above. The mitochondrial membrane potential was measured by flow cytometry using a fluorescent cationic dye according to the vendor's protocol (DePsipher ™, R &D System Inc.) as described before [23]. DePsipher has the property of aggregating upon membrane polarization forming an orange-red fluorescent (absorption/emission 585/590 nm) compound. If the membrane potential is reduced, the dye cannot access the transmembrane space and remains in its green fluorescent (510/527 nm) monomeric form.

### Measurement of ATP level

HepG2 cells were treated with different concentrations of ASA alone or after manipulation of GSH concentration by BSO with or without NAC as described above. The ATP content in the cell lysate was determined using ATP Bioluminescent cell assay kit according to the manufacturer's suggestion (Sigma, St Louis, MO) and samples were read using the TD-20/20 Luminometer (Turner Designs, Sunnyvale, CA).

### Measurement of mitochondrial respiratory functions

Freshly isolated mitochondria (5 µg protein) from GSH depleted with or without NAC treated HepG2 cells were suspended in 1.0 ml of 20 mM KPi buffer, pH 7.4, in the presence of the detergent, lauryl maltoside (0.2%). NADH-ubiquinone oxidoreductase (complex I), and cytochrome c oxidase (complex IV) were measured using the substrates coenzyme Q2 and reduced cytochrome c, respectively, by the methods of Birch-Machin and Turnbull [26] as described before [23].

Mitochondrial aconitase activity was measured by NADPH coupled conversion of citrate to isocitrate in the presence of isocitrate dehydrogenase using the Bioxytech Aconitase-340 assay kit. Aconitase activity was calculated according to the rate of formation of NADPH at 340 nm.

### Measurement of the expression of proteins by SDS-PAGE/Western Blotting and immunofluorescence microscopy

Proteins (50 µg) from the different sub cellular fractions of control and treated cells were separated on 12% SDS-PAGE and electrophoretically transferred onto nitrocellulose paper by Western blotting using the standard procedures of Laemmli [27] and Towbin et al. [28] as described before [23]. The immunoreacting protein bands were visualized after interacting with primary antibodies against cytochrome c, caspase-3, PARP, GSTA4-4 and Bcl-2. The expression of β-actin and Tom-40 proteins were used as respective house-keeping loading controls for post-mitochondrial supernatant and mitochondrial fractions. Densitometric analysis of the immunoreactive protein bands was performed using the gel documentation system (Vilber Lourmat, France) and expressed as relative intensity (R.I) compared to the untreated control.

For immunofluorescence microscopy, HepG2 cells (2.5×10^3^) were grown on cover slips in 6-well plates and were treated with desired doses of ASA after treatment with BSO with or without NAC. Cells were then fixed in 1% paraformaldehyde in PBS and permeabilized with 0.1% Triton X-100 in PBS for 10 mins. Cells were then blocked with 5% goat serum for 1 h at R.T and stained with 1∶300 dilution of anti-GST A4-4 polyclonal antibody overnight at 4°C, followed by incubation with FITC-conjugated secondary antibody for 1 h at room temperature as described before [29]. Cover slips were then mounted on to glass slides and examined and photographed using an Olympus microscope with epifluorescence optics and interference filters.

### Statistical analysis

Values shown are expressed as mean ± SEM of three determinations. Statistical significance of the data was assessed by analysis of variance (ANOVA) followed by Tukey's post-hoc test and p values≤0.05 were considered statistically significant.

## Results

### Aspirin-induced alterations in GSH pool

**Figure 1:**
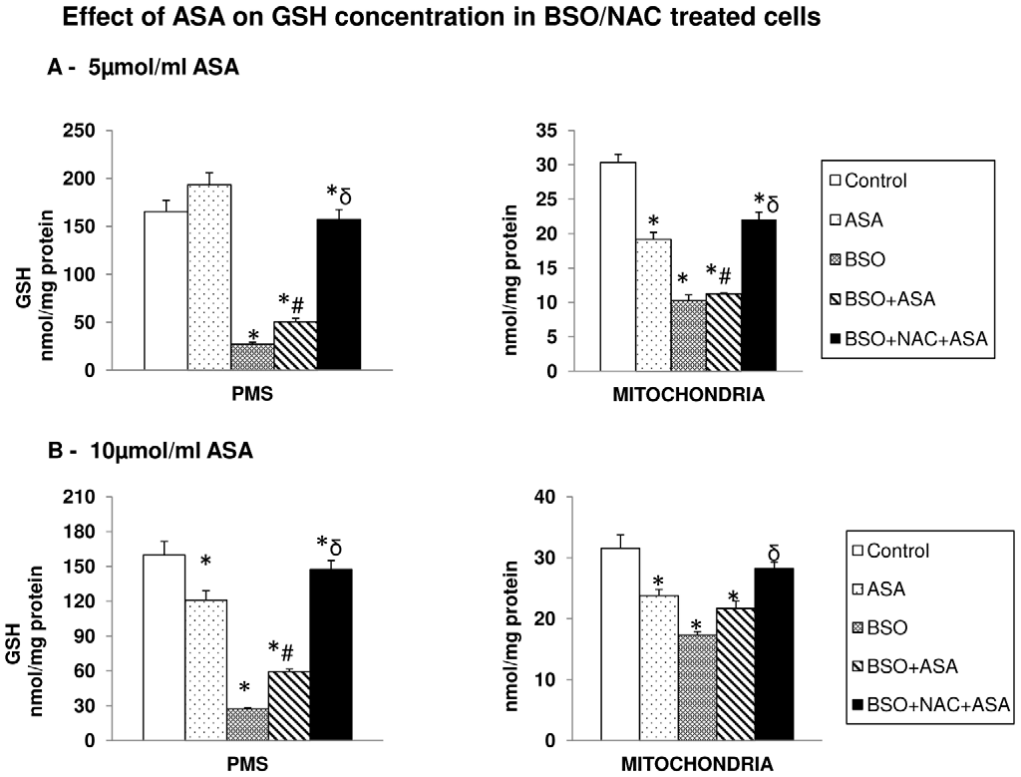
Aspirin-induced alterations in the GSH pool. HepG2 cells were treated with 5 µmol/ml (Figure 1A) and 10 µmol/ml (Figure 1B) of ASA for 48 h and GSH levels in the mitochondria and post mitochondrial supernatant (PMS) were measured by enzymatic method as described in the Materials and Methods. Results are expressed as mean ± SEM of three determinations. Figure also represents measurement of GSH levels after treatment of cells with 100 µM BSO for 18 h alone or after treatment with 10 mM NAC for 2 h prior to the ASA treatment. The values are expressed as mean ± SEM of three determinations. Asterisks (*) indicate significant difference (P≤0.05) from control values, # indicate significant difference (P≤0.05) from ASA treated group and δ indicate significant difference (P≤0.05) as compared to BSO+ASA treated group.

Our recent study on dose- and time-dependent effects of ASA has shown that the low therapeutic doses of ASA exhibited no significant effect on cell viability. ASA treatment at 5 µmol/ml caused only 10–15% inhibition in cell viability while 10 µmol/ml ASA caused about 30–40% loss in cell viability in 48 hours [23]. Selection of doses and time points in the present study was based on these results and data from other published reports [8], [12], [13]. In the present study, we have shown that low dose of ASA (5 µmol/ml) caused no significant effect on GSH pool in the PMS (Figure 1A) in HepG2 cells while 10 µmol/ml ASA caused a significant reduction (Figure 1B). On the other hand, a marked reduction (30–40%) in the mitochondrial GSH pool was observed under these conditions (Figure 1A and Figure 1B). BSO treatment, which inhibits the rate limiting enzyme for GSH synthesis, resulted in almost 80% reduction in extra mitochondrial GSH concentration compared to about 50% reduction in the mitochondrial GSH pool suggesting that cytosolic GSH is more sensitive to BSO compared to mitochondrial GSH. ASA treatment did not reduce the total GSH pools any further. NAC treatment, however, resulted in partial recovery of mitochondrial GSH pool while almost a complete recovery in the cytosolic GSH pool was observed. This suggests an increased sensitivity of mitochondrial GSH towards ASA treatment.

### Aspirin-induced apoptosis in HepG2 cells

**Figure 2:**
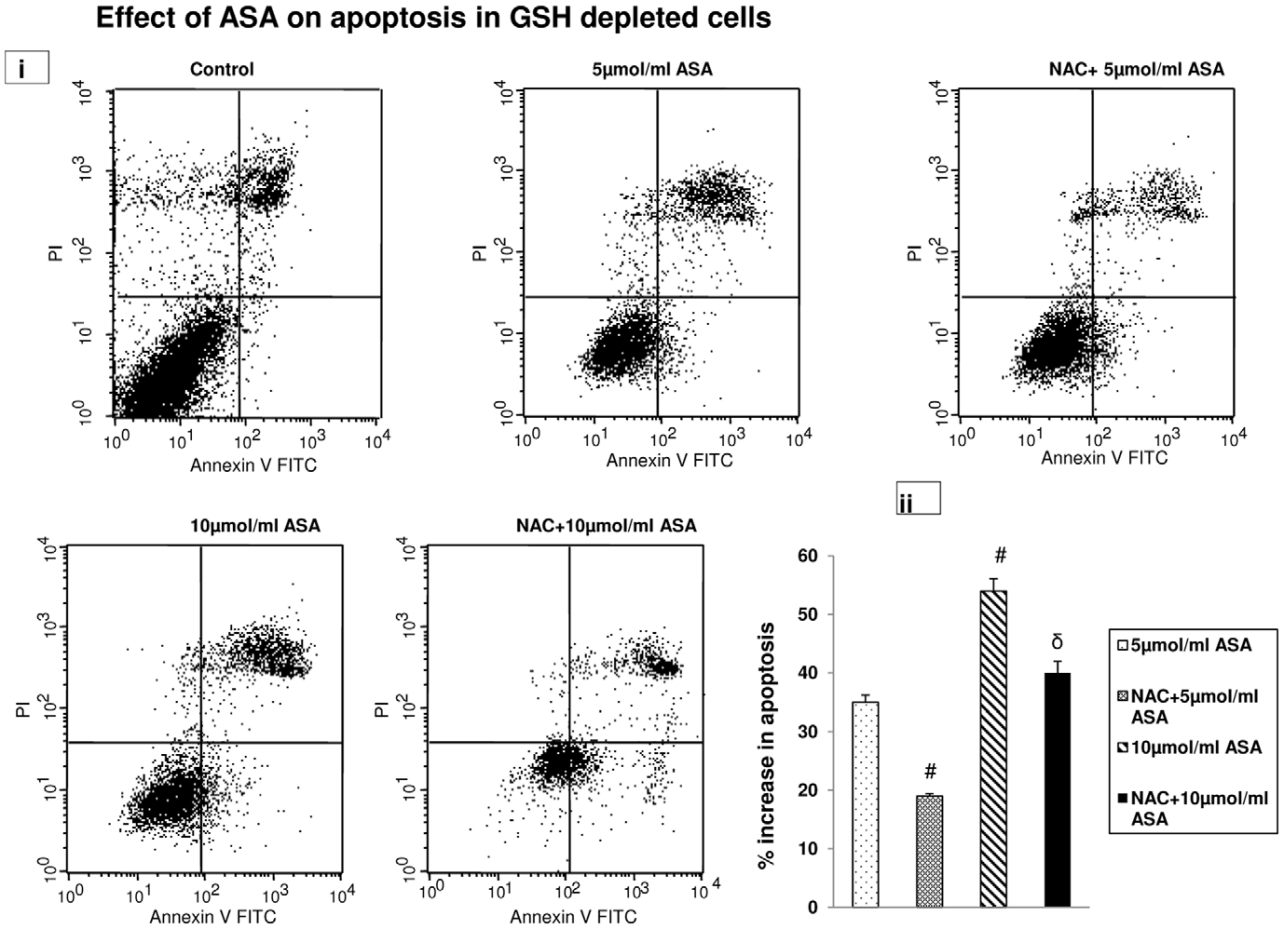
Aspirin-induced apoptosis. Apoptosis in GSH depleted and NAC treated HepG2 cells were measured after ASA treatment using Flow cytometry (Figure 2i) as described in the vendor's protocol using the Becton Dickinson FACScan analyzer. Apoptotic cells were estimated by the percentage of cells that stained positive for Annexin V-FITC. Histogram showing the percentage increase in apoptosis after ASA and NAC treated GSH-depleted cells is shown in Figure 2ii. # indicate significant difference (P≤0.05) from 5 µmol/ml ASA treated group and δ indicate significant difference (P≤0.05) as compared to 10 µmol/ml ASA treated group.

As shown in Figure 2 (i), the apoptosis assay by flow cytometry has demonstrated that increasing doses of ASA treatment in GSH- depleted cells caused increased apoptosis in HepG2 cells. When compared with the control (which exhibited about 14% spontaneous apoptosis), low dose (5 µmol/ml) of ASA showed a 49% increase in apoptosis in GSH-depleted cells while 10 µmol/ml ASA caused 68% increase in apoptosis after 48 h. NAC treatment, however, caused only moderate recovery of aspirin-induced apoptosis in HepG2 cells suggesting additional factor(s), other than altered GSH pool, which could play a role in inducing apoptosis by ASA. A histogram showing net increase in apoptosis after subtraction of cell death caused by GSH depletion in control cells is shown in Figure 2(ii).

### Aspirin-induced ROS production

**Figure 3:**
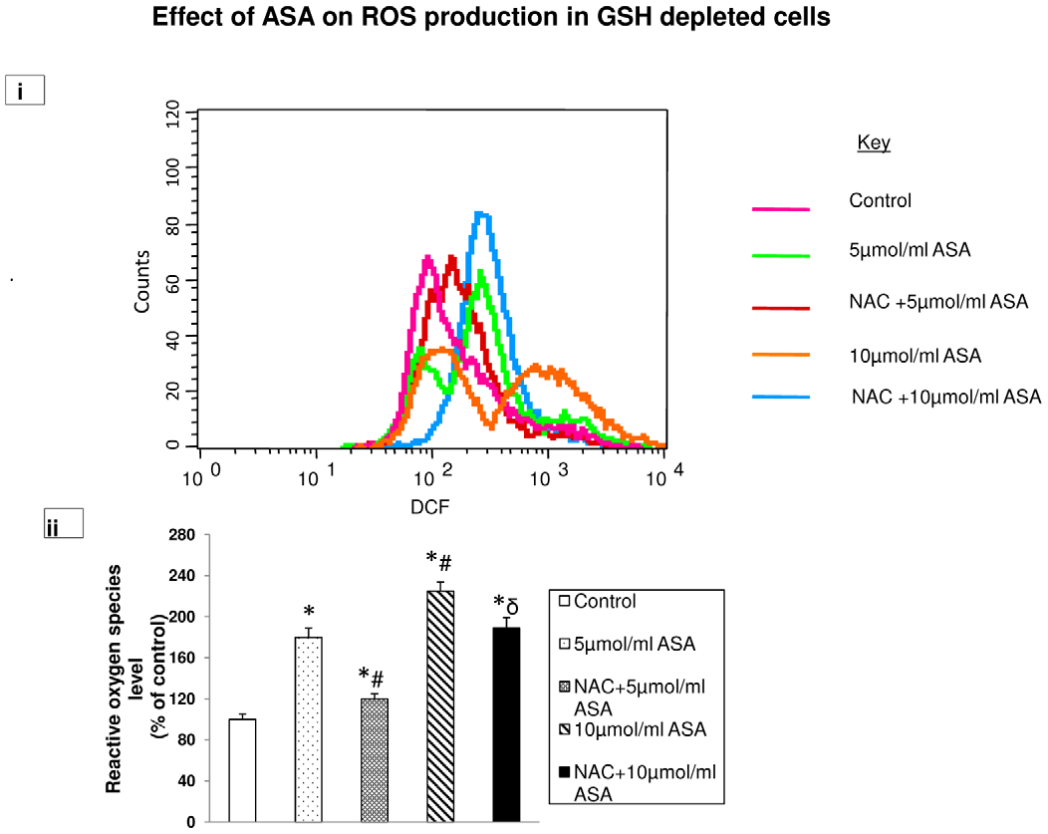
Aspirin-induced ROS production. Intracellular production of ROS was measured using DCFDA and fluorescence was measured by flow cytometry using Cell Quest software as described in the Materials and Methods. ROS was measured in GSH depleted HepG2 cells treated with different concentrations of aspirin and also after NAC treatment (Figure 3i). Result expressed is a typical representation of three determinations. % change in ROS production from a typical histogram is presented in Figure 3ii. Asterisks indicate significant difference (P≤0.05) from control values, # indicate significant difference (P≤0.05) from 5 µmol/ml ASA treated group and δ indicate significant difference (P≤0.05) as compared to 10 µmol/ml ASA treated group.

Using DCFDA as a fluorescent probe, we observed a significant increase (almost 2–2.5 fold) in ROS production in GSH-depleted cells after 5 µmol/ml and 10 µmol/ml ASA treatments (Figure 3 i and ii). NAC treatment brought the level of ROS close to control level in 5 µmol/ml ASA treated cells while only partial recovery was observed in cells treated with 10 µmol/ml ASA. These results further suggest that GSH plays only a partial role in the regulation of ROS production in ASA treated HepG2 cells.

### Aspirin-induced DNA fragmentation

**Figure 4:**
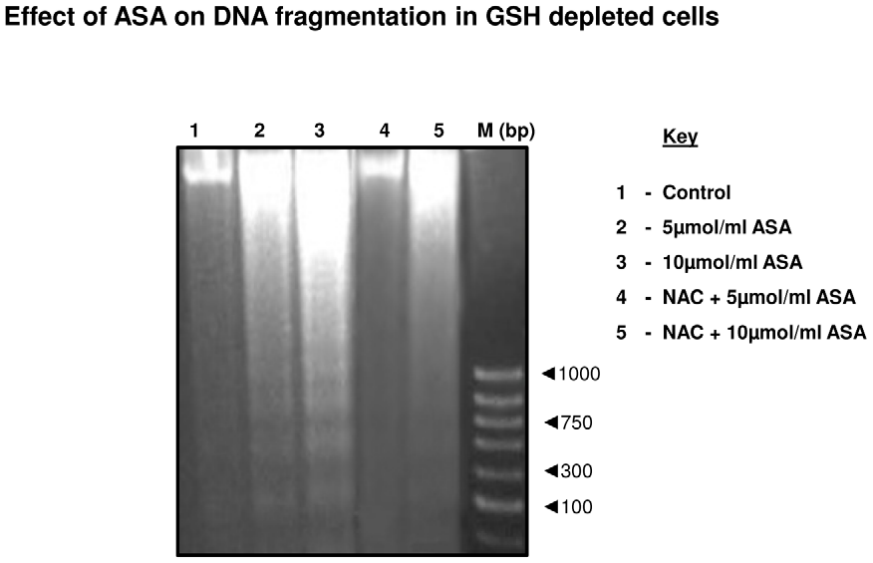
Aspirin-induced DNA fragmentation. DNA was isolated from GSH-depleted and NAC treated cells after treatment with different doses of ASA as described in the Materials and Methods. DNA fragmentation was studied using 1.5% agarose gel electrophoresis and visualized by ethidium bromide staining. A 1000 base pair DNA ladder was used as marker. A typical result from three experiments has been shown here.

As shown in Figure 4, increased DNA fragmentation in GSH-depleted HepG2 cells was observed after ASA treatment (lane 2 and 3). NAC treatment appears to have partially protected the breakdown of DNA which was more apparent with 5 µmol/ml (lane 4) compared to 10 µmol/ml ASA (lane 5) treated cells.

### Aspirin-induced alterations in GSH metabolism

**Figure 5:**
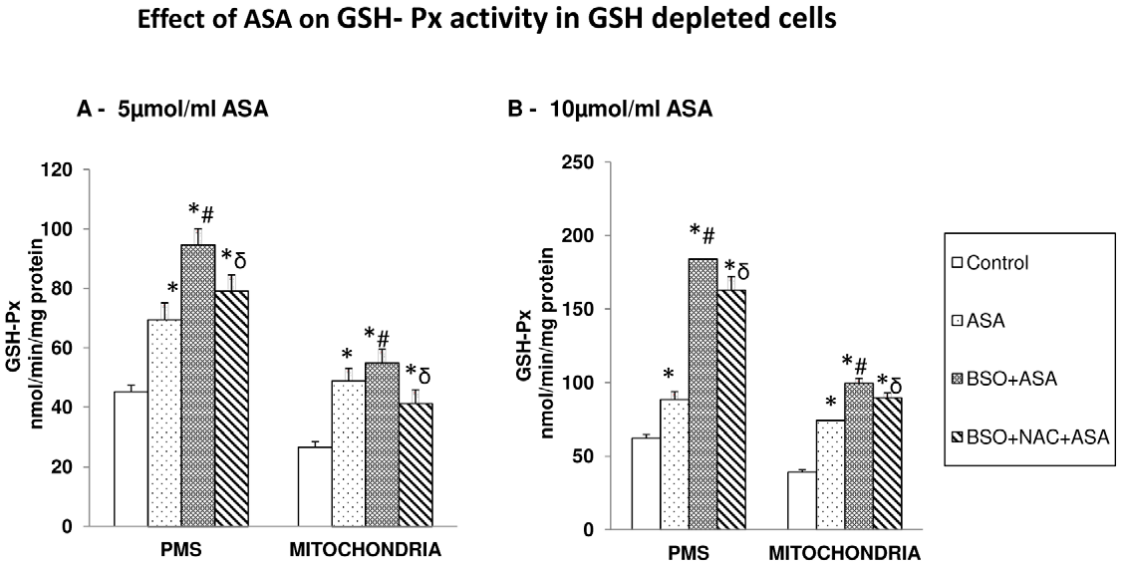
Aspirin-induced alterations in GSH Peroxidase. GSH-peroxidase (Figures 5A, B) was measured in GSH-depleted cells after treatment with different concentrations of ASA and NAC as described in the Materials and Methods. The values are expressed as mean ± SEM of three determinations. Asterisks indicate significant difference (P≤0.05) from control values, # indicate significant difference (P≤0.05) from ASA treated group and δ indicate significant difference (P≤0.05) as compared to BSO+ASA treated group.

GSH-Px activity in HepG2 cells was significantly increased after ASA treatment (Figure 5A and B).The enzyme activity was further increased (40%) in GSH-depleted- 5 µmol/ml ASA treated cells and almost 2-fold in GSH-depleted 10 µmol/ml ASA treated cells in the PMS while mitochondria showed only 15–30% increased activity in GSH-depleted cells. NAC treatment, however, showed only partial recovery of the enzyme activity.

**Figure 6:**
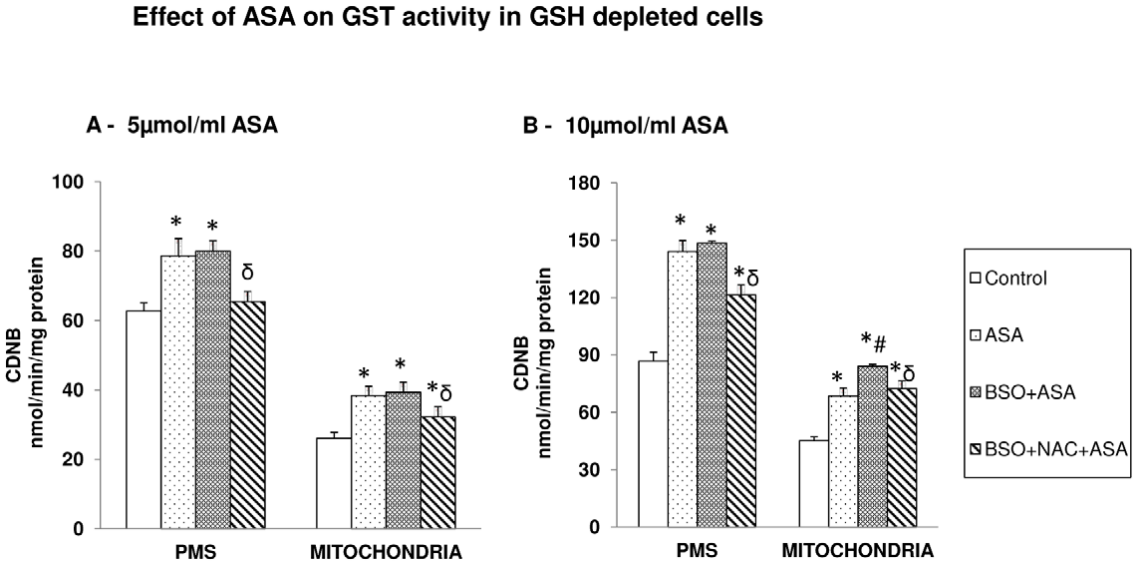
Aspirin-induced alterations in GST. GST activity (Figures 6 A, B) was measured in GSH-depleted cells after treatment with different concentrations of ASA and NAC using CDNB as substrate as described in the Materials and Methods. The values are expressed as mean ± SEM of three determinations. Asterisks indicate significant difference (P≤0.05) from control values, # indicate significant difference (P≤0.05) from ASA treated group and δ indicate significant difference (P≤0.05) as compared to BSO+ASA treated group.

Total GST activity using CDNB as substrate was significantly increased in HepG2 cells after ASA treatment (Figure 6A and 6B). The enzyme activity was further increased in GSH depleted cells treated with ASA. NAC treatment brought the enzyme activity close to control values in 5 µmol/ml ASA treated cells (Figure 6A) but not in 10 µmol/ml ASA treated cells, where the enzyme activity remained significantly higher (Figure 6B). These results were further confirmed by SDS-PAGE and immunofluorescence studies on the expression of GST A4-4 protein using isoenzyme specific antibody as described later.

### Aspirin-induced increase in LPO

**Figure 7:**
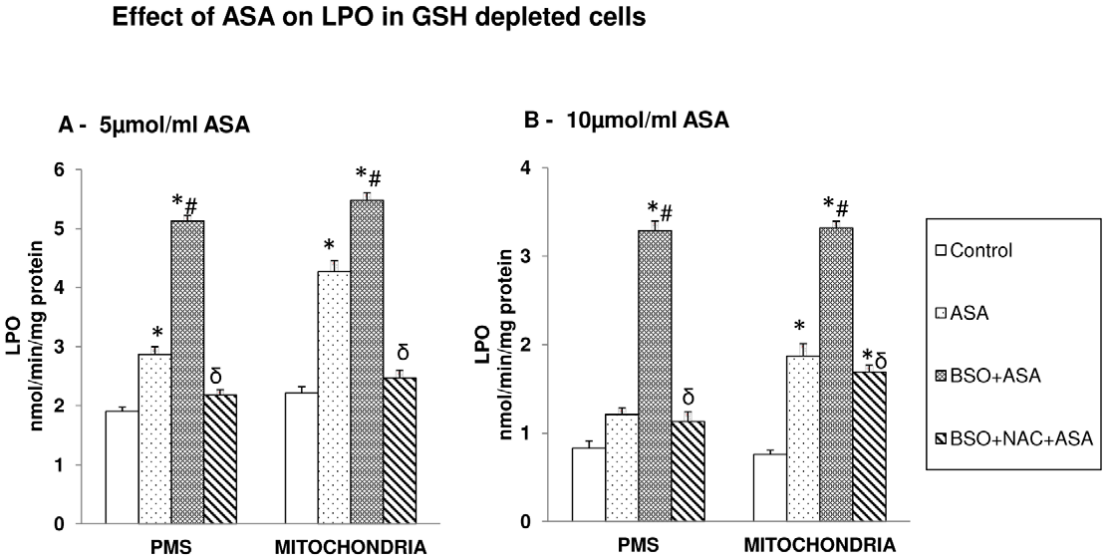
Aspirin-induced LPO. Membrane LPO was determined in GSH-depleted cells after treatment of ASA and NAC (Figures 7A, B) as described in the Materials and Methods. The values are expressed as mean ± SEM of three determinations. Asterisks indicate significant difference (P≤0.05) from control values, # indicate significant difference (P≤0.05) from ASA treated group and δ indicate significant difference (P≤0.05) as compared to BSO+ASA treated group.

ASA treatment caused a significant increase in LPO (Figure 7A, B). It was further increased in GSH-depleted cells treated with ASA. NAC treatment resulted in complete recovery of LPO in 5 µmol/ml ASA- treated cells (Figure 7A) while 10 µmol/ml ASA treated cells exhibited only partial recovery (Figure 7B).

### Aspirin-induced caspase-3 activation

**Figure 8:**
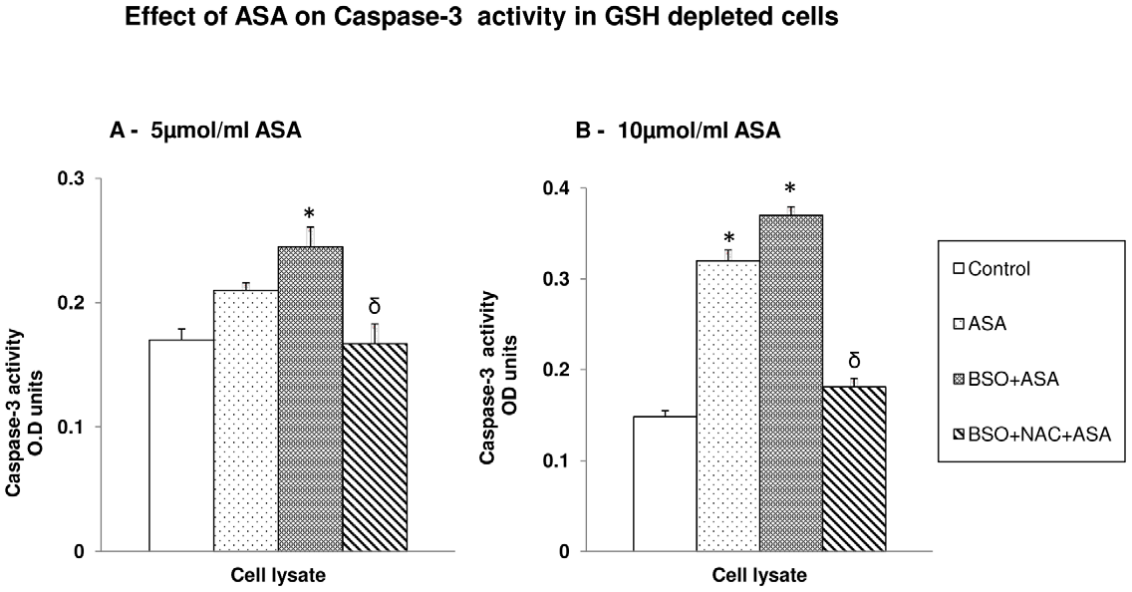
Aspirin induced caspase-3 activity. Caspase-3 activity in HepG2 cells treated with aspirin and after manipulation of GSH levels by BSO and NAC was measured using DEVD peptide conjugated to p-nitroanaline as described in the vendor's protocol (Figures 8A, B). The values are expressed as mean ± SEM of three determinations. Asterisks indicate significant difference (P≤0.05) from control values while δ indicate significant difference (P≤0.05) as compared to BSO+ASA treated group.

ASA treatment caused a significant increase in caspase-3 activity with 10 µmol/ml ASA compared to 5 µmol/ml ASA (Figure 8 A, B). The enzyme activity remained markedly increased after ASA treatment in GSH depleted cells. NAC treatment, on the other hand, brought the enzyme activity to the level of control untreated cells. These results have further confirmed the increased apoptosis and DNA fragmentation observed (Figure 2 and 4) after ASA treatment in GSH depleted cells and further established the protective effect of NAC from drug induced apoptosis.

### Aspirin-induced reduction in mitochondrial membrane potential (MMP)

**Figure 9:**
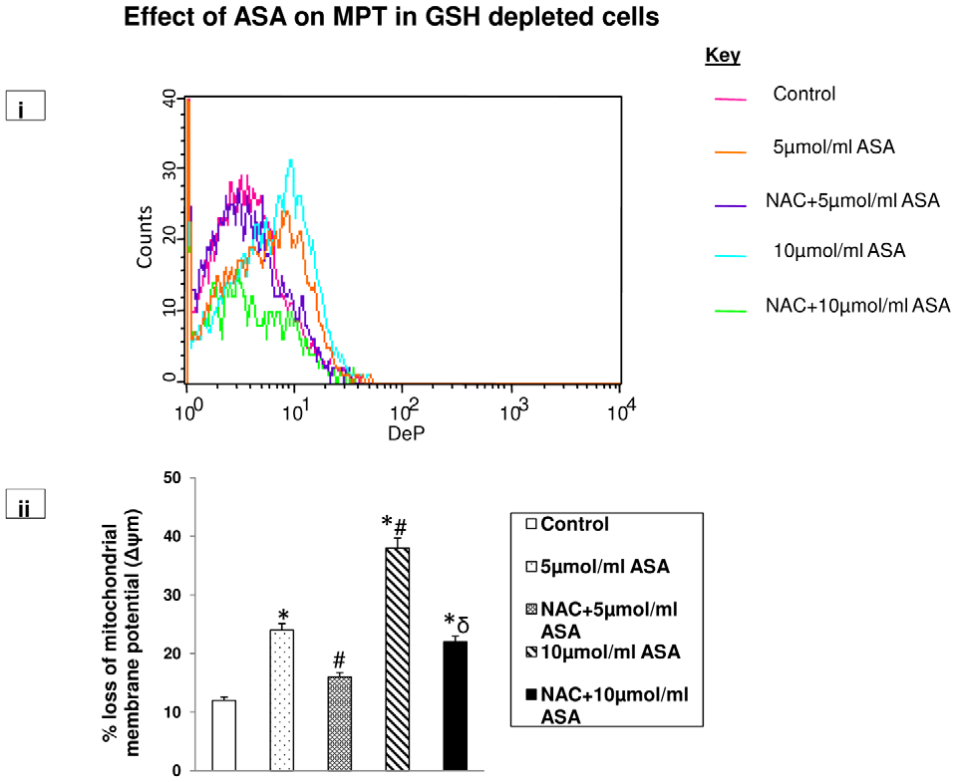
Aspirin-induced disruption in mitochondrial membrane potential. GSH-depleted HepG2 cells were treated with different concentrations of aspirin and membrane potential was measured using a cationic fluorescent dye as described in the Materials and Methods. Mitochondrial membrane potential was also measured after manipulation of GSH levels by NAC treatment. Figure 9i is a representative of three individual experiments. Figure 9ii is a typical histogram showing % loss of mitochondrial membrane potential. Asterisks indicate significant difference (P≤0.05) from control values, # indicate significant difference (P≤0.05) from 5 µmol/ml ASA treated group and δ indicate significant difference (P≤0.05) as compared to 10 µmol/ml ASA treated group.

A significant loss (2–4 fold) in mitochondrial membrane potential was observed after ASA treatment in GSH depleted cells (Figure 9 i and ii).The effect of ASA appeared to be dose dependent. NAC treated cells, however, showed partial recovery from ASA-affected MMP, suggesting only partial protection of mitochondrial membrane permeability by GSH pool.

### Aspirin-induced mitochondrial dysfunction

#### Effect on ATP level

**Figure 10:**
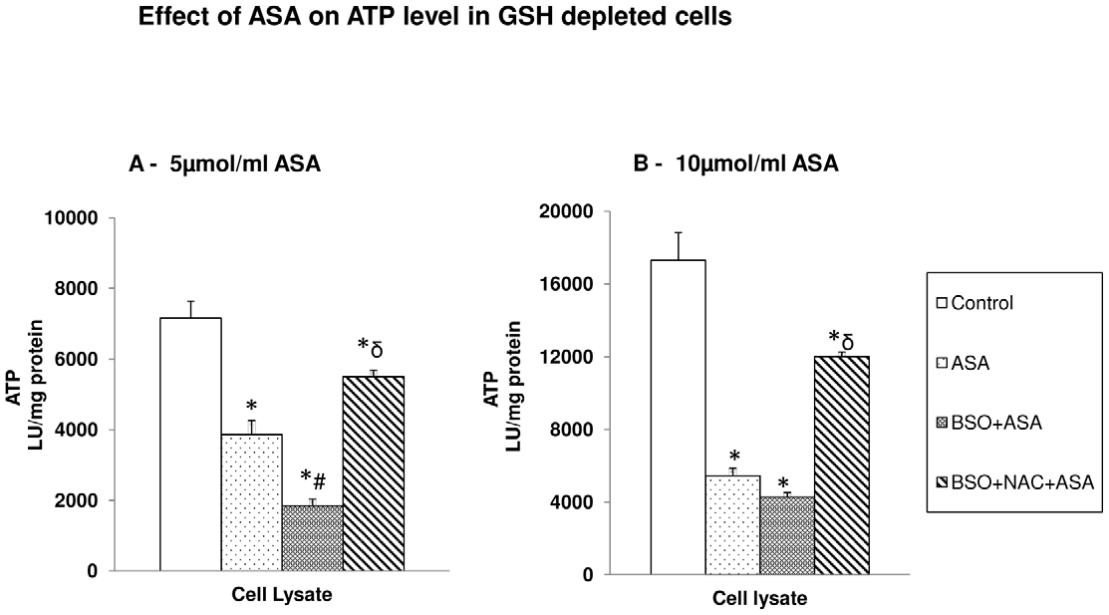
Aspirin-induced alteration in ATP level. HepG2 cells were treated with different concentrations of aspirin after BSO/NAC treatment and ATP content was measured in the total cell lysate (Figures 10A, B) using the ATP Bioluminescent somatic cell assay kit as described in the Materials and Methods. The values are expressed as mean ± SEM of three determinations. Asterisks indicate significant difference (P≤0.05) from control values, # indicate significant difference (P≤0.05) from ASA treated group and δ indicate significant difference (P≤0.05) as compared to BSO+ASA treated group.

ASA caused a significant decrease in cellular ATP level with increasing dose (Figure 10A, B). GSH depletion in ASA treated cells further reduced the level of ATP. NAC treatment exhibited partial recovery from ATP depletion in GSH-depleted cells.

#### Effect on the mitochondrial matrix enzyme, aconitase

**Figure 11:**
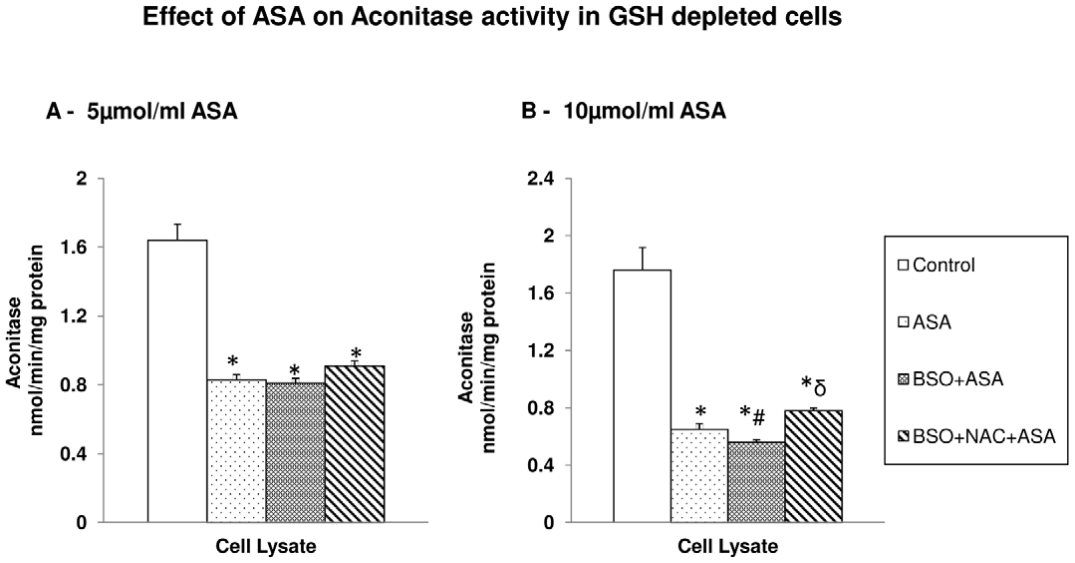
Aspirin-induced alteration in aconitase activity. The HepG2 cells were treated with different concentrations of aspirin and aconitase activity was measured after the manipulation of GSH level by BSO or NAC (Figures 11A, B) as described in the Materials and Methods. The values are expressed as mean ± SEM of three determinations. Asterisks indicate significant difference (P≤0.05) from control values, # indicate significant difference (P≤0.05) from ASA treated group and δ indicate significant difference (P≤0.05) as compared to BSO+ASA treated group.

The mitochondrial matrix enzyme, aconitase, was also markedly inhibited by ASA treatment. The effect on the enzyme activity appeared to be dose dependent (Figure 11A, B). GSH depletion prior to ASA treatment resulted in further inhibition of aconitase activity. NAC treatment, however, showed no recovery in GSH depleted treated cells.

#### Effect of ASA on respiratory functions

**Figure 12:**
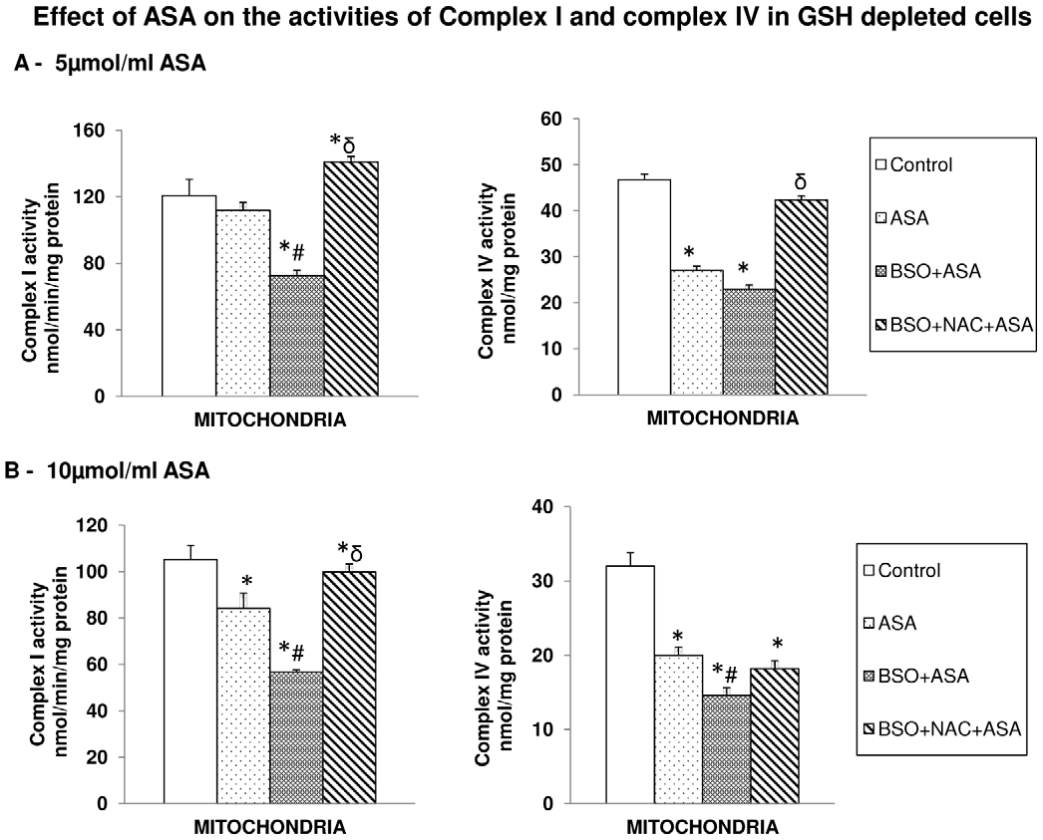
Aspirin-induced alterations in mitochondrial Complex I and Complex IV activities. Freshly isolated mitochondria from control and aspirin treated HepG2 cells after BSO/NAC treatments were used to assay Complex I and cytochrome c oxidase activity as described in the Materials and Methods. (Figures 12A, B). The values are expressed as mean ± SEM of three determinations. Asterisks indicate significant difference (P≤0.05) from control values, # indicate significant difference (P≤0.05) from ASA treated group and δ indicate significant difference (P≤0.05) as compared to BSO+ASA treated group.

Only a marginal inhibition of Complex I activity was observed after ASA treatment. On the other hand, a significant inhibition (∼50%) in Complex IV (cytochrome c oxidase) activity was observed after ASA treatment (Figure 12 A, B). Complex I activity inhibition by ASA was more pronounced (almost 50%) in GSH-depleted cells while NAC treatment caused complete recovery of the enzyme activity. Similarly, the inhibitory effect of ASA on Complex IV activity was more pronounced in GSH-depleted cells. Unlike its effect on Complex I activity, NAC could not recover cytochrome c oxidase activity in GSH-depleted cells treated with 10 µmol/ml ASA suggesting higher sensitivity of the enzyme towards ASA/GSH-depletion.

#### Aspirin-induced expression of apoptotic markers

**Figure 13:**
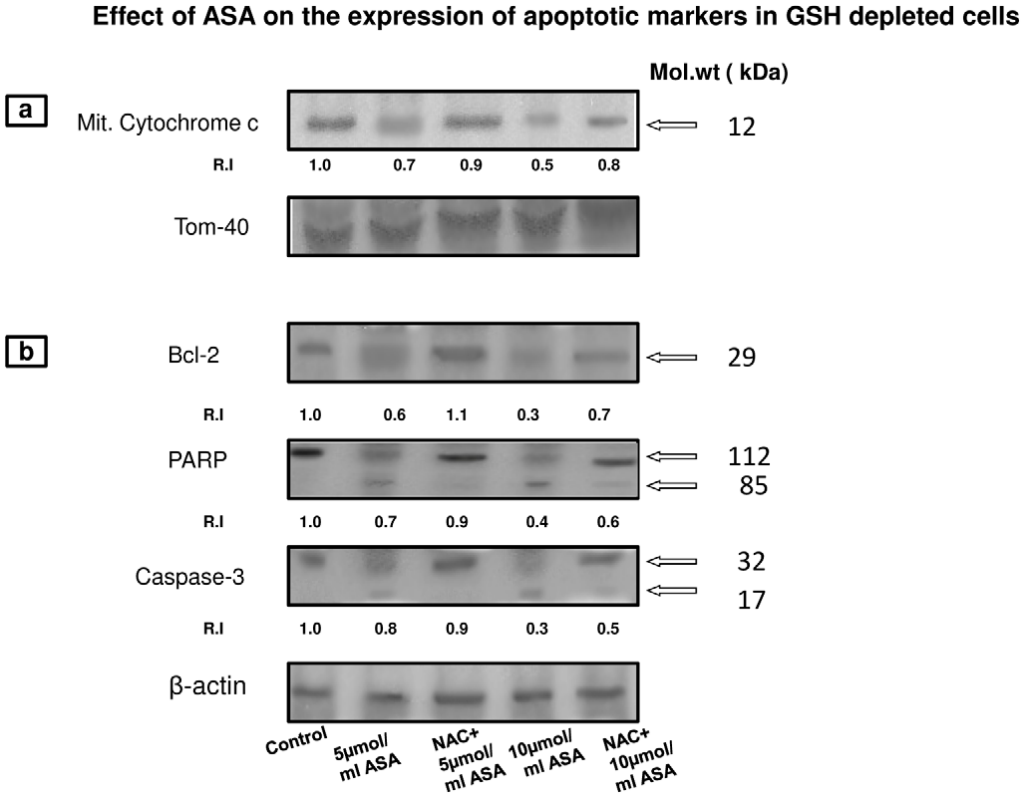
Expression of apoptotic markers. Proteins (50 µg) from PMS or mitochondrial extract from ASA/NAC treated GSH-depleted HepG2 cells were separated on 12% SDS-PAGE and transferred on to nitrocellulose paper by Western blotting as described in the Materials and Methods. The visualization of cytochrome c (Figure 13a) and Bcl-2, PARP and caspase-3 (Figure 13b) was made by using specific antibodies against these proteins. Beta-actin and Tom-40 were used as respective loading controls for post-mitochondrial supernatant and mitochondria. Figure is a typical result obtained from three independent experiments. The quantitation of proteins bands are expressed as relative intensity (R.I) of the upper band using expression of proteins in control untreated cells as 1.0. Molecular weight markers (kDa) are indicated by arrows.

As shown in Figure 13a, ASA treatment in GSH-depleted cells reduced the level of cytochrome c in the mitochondria. The translocation of cytochrome c from the mitochondria was more apparent after treatment with 10 µmol/ml ASA. The release of cytochrome c from the mitochondria, in turn, resulted in activation of caspase-3 (Figure 13b). This in turn, was accompanied by activation of PARP, suggesting the activation of DNA repair mechanism and apoptotic signals in ASA treated cells. The expression of the antiapoptotic protein, Bcl-2 was also inhibited in GSH-depleted ASA treated cells. All these results suggest activation of the apoptotic process in HepG2 cells after ASA treatment. NAC treatment, however, resulted in partial recovery of the apoptotic markers described above suggesting partial protection from the deleterious effects of the drug.

#### Immunocytochemical studies on the expression of GSTA4-4

**Figure 14:**
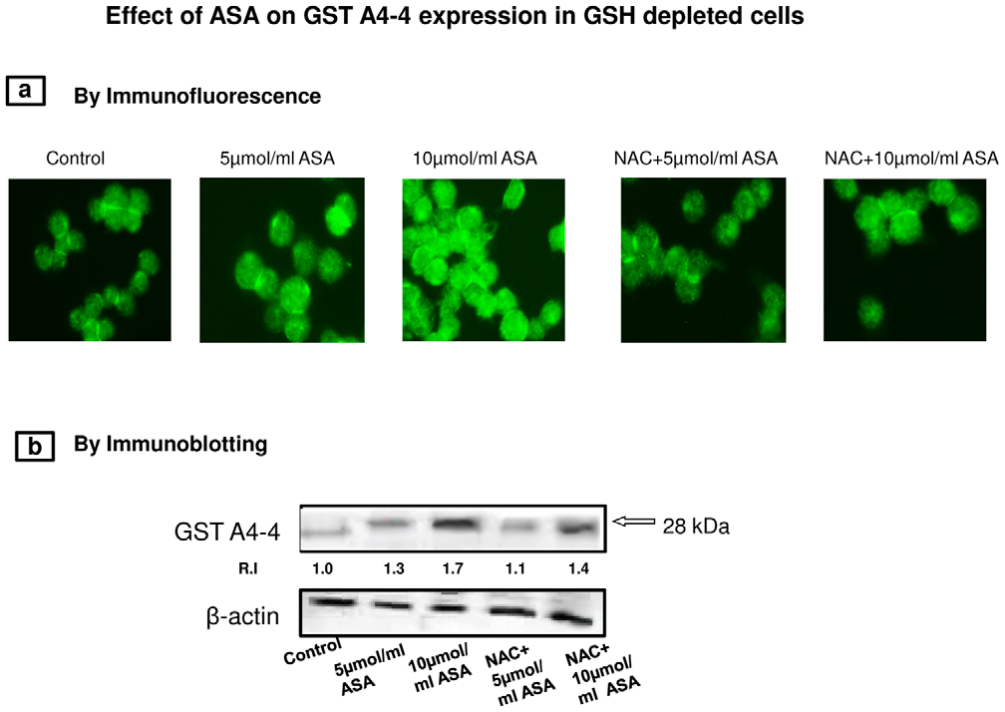
Immunocytochemical localization of GST A4-4. HepG2 cells were grown on cover slips and treated with ASA after GSH depletion with or without NAC as described in the Materials and Methods. Cells were then fixed in 1% paraformaldehyde and permeabilised in 0.1% Triton X-100. Cells were incubated with 1∶300 dilution of GST A4-4 antibody followed by FITC-conjugated secondary antibody. Cover slips were then examined and photographed using an Olympus fluorescent microscope (Figure 14a). Immunoblotting was carried out using GSTA4-4 specific polyclonal antibody (Figure 14b). The quantitation of the protein bands are expressed as relative intensity (R.I) compared to the control untreated cells as 1.0. Molecular weight marker (kDa) is indicated by an arrow. β-actin was used as a loading control.

Figure 14a shows an increased expression (immunofluorescence) of GSTA4-4, an oxidative stress specific GST isoenzyme, in HepG2 cells after ASA treatment.NAC treatment brought the enzyme activity close to control values in 5 µmol/ml ASA treated cells but not in 10 µmol/ml ASA treated cells, where the enzyme expression remained significantly higher. The increased expression of GSTA4-4 was also confirmed by Western blot analysis as shown in Figure 14b.

## Discussion

Epidemiological and experimental studies have reported anti-inflammatory, anticancer and antidiabetic effects of NSAIDs. The molecular mechanism(s) by which aspirin and other NSAIDs exert their anticancer effects, however, is still not clear. In addition to the inhibition of arachidonic acid metabolism by COX inhibition, other possible mechanisms for the multiple effects of ASA include increased ROS production, alterations in mitochondrial function, cell signaling and induction of apoptosis [7], [19], [30], [31], [32], [33]. Although in this study and in our previous study [23] on HepG2 cells, we have used ASA to elucidate COX-independent mechanisms of drug action, there are reports that suggest that salicylate, an active metabolite of aspirin, has more or less similar mechanisms of action on mitochondrial-induced apoptosis [30], [31]. We have reported previously that ASA induces cell cycle arrest, mitochondrial dysfunction and oxidative stress in HepG2 cells [23]. The increased apoptosis observed was presumably initiated by the altered mitochondrial membrane potential causing release of cytochrome c and activation of the intrinsic apoptotic pathway. ASA also caused dose and time-dependent increase in ROS production and decrease in GSH pool. Mitochondrial GSH pool homeostasis has been shown to be closely coupled with Bcl-2 translocation and apoptosis [34], [35], [36]. Our study had shown a marked initial depletion in mitochondrial GSH pool by aspirin. However, it was not clear how ASA exerts its multiple effects in oxidative stress conditions when cellular GSH pool is altered (depleted or enhanced). Therefore, in the present study, we investigated the effect of ASA on HepG2 cells after treating the cells with BSO, a GSH synthesis inhibitor and NAC, a GSH synthesis precursor. We observed that GSH depletion in HepG2 cells by BSO selectively inhibited cytosolic GSH pool in comparison to the mitochondrial GSH pool. This is presumably due to the fact that mitochondria do not have a GSH synthesizing enzyme system and depend upon the energy/transporter-dependent transfer of GSH from the cytosol [37]. Our results in the present study show that ASA-induced mitochondrial dysfunction and oxidative stress were further augmented in GSH-depleted cells, while NAC treatment attenuated the effects of aspirin in GSH-depleted cells. Our study has also shown that the effect of NAC on ASA treatment in GSH-depleted cells was selective in terms of recovery of GSH metabolism and oxidative stress in different cellular compartments and on mitochondrial functions and apoptosis. The recovery of respiratory Complex I activity and membrane potential was significant after NAC treatment while that of ATP synthesis and activities of Complex IV and the matrix enzyme, aconitase was negligible in GSH-depleted cells. ROS production was high in GSH-depleted cells even after NAC treatment suggesting recovery in membrane potential after NAC treatment may partially be independent of ROS production and GSH pool in mitochondria. GSH depletion in HepG2 cells augmented LPO and NAC treatment resulted in partial protection from LPO suggesting the protective role of mitochondrial GSH metabolism in membrane lipid peroxidation. Increased GSH-Px activity observed after ASA treatment might be a protective mechanism to increase ROS clearance and protect vital cellular functions from oxidative stress associated complications. Similarly, GSH-conjugation/detoxification (including ROS) activity by GST, which also has GSH-peroxidase activity, is increased by ASA in GSH-depleted cells. We have previously shown increased expression and activities of GSTs in oxidative stress conditions. We have also shown that GSTA4-4 was specifically induced in oxidative stress conditions [25], [29], [37], [38]. In the present study also, using SDS-PAGE and immunofluorescence microscopy, we have confirmed the increased expression of GSTA4-4 in GSH-depleted cells treated with ASA. NAC treatment resulted in partial decrease in GSTA4-4 expression. NAC treatment also resulted in partial protection of apoptosis as observed by the reduced cytochrome c release from mitochondria and reduced activation of caspase-3 and PARP hydrolysis.

In confirmation of our previous study [23], in the present study also, we observed that alterations in the GSH pool altered the mitochondrial bioenergetics in ASA treated cells. ASA caused a marked decrease in ATP level in GSH-depleted cells. The reduced ATP level in ASA treated cells was dose dependent. The decrease in ATP production by ASA was accompanied by inhibition in the activities of respiratory chain enzymes particularly, cytochrome c oxidase (Complex IV) and the mitochondrial matrix enzyme, aconitase. These enzymes determine the rate of mitochondrial oxygen utilization, ROS production, oxidative stress and ATP synthesis [15], [16], [39], [40], [41], [42], [43], [44]. Recently, it has been shown that mitochondrial GSH pool and expression of antiapoptotic protein Bcl-2 are directly involved in the regulation of mitochondrial membrane potential, redox and respiratory functions [36], [45]. Our results have also shown a decrease in Bcl-2 expression after ASA treatment which was augmented in GSH depleted cells and attenuated after NAC treatment. The precise mechanisms by which GSH depletion regulates apoptosis are, however, not clear. Studies have suggested that HepG2 cells undergoing apoptosis have greater loss of intracellular GSH due to increased export of GSH to extracellular space [46]. A recent study has also suggested that depletion of GSH regulates apoptosis independent of excessive ROS production [47]. Therefore, maintenance of intracellular GSH levels during apoptosis provides protection to the cell by multiple mechanisms. NAC, a thiol antioxidant, is increasingly used in clinical trials of chemotherapy and as a chemoprotectant in drug-induced toxicity [48]. In our study, the marked reduction of ATP level in GSH depleted cells was attenuated by NAC treatment. This observation was further supported by the recovery in Complex I activity in ASA treated cells in the presence of NAC. However, NAC treatment could not completely recover all the mitochondrial respiratory functions as the activities of cytochrome c oxidase and aconitase remained inhibited in the drug treated cells even after NAC treatment. This suggests that mitochondrial GSH is selectively involved in regulating the activities of the respiratory complexes. The inhibitory effect of ASA on cytochrome c oxidase and aconitase activities was more apparent in GSH depleted cells. Our previous studies, using in vitro cell culture as well as in vivo animal models have also demonstrated that enzymes cytochrome c oxidase (Complex IV) and aconitase are extremely sensitive to mitochondrial-ROS induced oxidative stress in comparison to other complexes [23],[38],[42],[43]. This differential sensitivity of respiratory complexes might be attributed to their responses towards oxidative stress, ROS, NO, and glutathionylation of proteins. Reversible glutathionylation of Complex I in response to oxidation of mitochondrial GSH pool has been shown to increase ROS formation [49]. Recovery of Complex I activity by NAC in our study might therefore be due to the decrease in oxidative glutathionylation of the protein. On the other hand, Complex IV activity has been reported to be reversibly inhibited by NO that increases superoxide production in mitochondria [50], [51]. Recent studies have shown that increased ROS production promotes reversible uncoupling of NO synthase (NOS) and shifting the enzyme activity to produce more ROS instead of NO [51], [52]. This uncoupling disrupts the cross-talk between ROS-generating enzymes and redox-cell signaling affecting the activities of respiratory complexes. Our recent study, on acetaminophen-induced toxicity in macrophages has also suggested that differential inhibition of mitochondrial respiratory enzymes is associated with altered ROS/NO homeostasis in mitochondria [53].Our recent publications have also discussed extensively the mitochondrial respiratory dysfunctions under increased oxidative stress conditions [23], [25], [38], [42], [43]. NAC does not seem to be involved in complete protection of mitochondrial respiratory functions in HepG2 cells from ASA-induced toxicity under these conditions. A similar observation with NAC treatment was also made when HepG2 cells were treated with acetaminophen [54]. These results clearly demonstrate that mitochondrial integrity and some of the key mitochondrial respiratory functions are indeed regulated by the antioxidant GSH pool and that they are potential selective targets for ASA- induced toxicity.

In summary, we have demonstrated that GSH depletion augmented the effects of ASA on mitochondrial functions and apoptosis in HepG2 cells by altering membrane potential, oxidative stress and activities of the respiratory chain enzymes. In addition, we have also shown that NAC plays a protective role, at least in part, in maintaining the GSH-dependent redox homeostasis and integrity of the mitochondrial functions. The expression of apoptotic signals also appears to be linked to alterations in the GSH pool. Therefore, a distinct outcome of the manipulation in GSH pool might be significant in therapeutic management of cancer and other inflammatory disorders. Our study suggests that manipulation of GSH homeostasis in HepG2 cells might have implications in ASA-induced sensitization of cancer cells which are potentially important in increasing the efficacy of anti-inflammatory chemotherapeutic drugs in inducing targeted apoptosis.
